# The ancient mitochondrial genome of Asiatic ibex (*Capra sibirica*) from Tangchaodun Ruins in Xinjiang, China, and its phylogenetic relationship

**DOI:** 10.1080/23802359.2025.2604864

**Published:** 2025-12-18

**Authors:** Guangjie Song, Xinyan Zhang, Xiaohong Yu, Dawei Cai

**Affiliations:** ^a^ School of Ethnic Studies, Xizang Minzu University, Xianyang, China; ^b^ Bioarchaeology Laboratory, Jilin University, Changchun, China

**Keywords:** Ancient DNA, high throughput sequencing, genetic continuity, genetic backgrounds, genetic architectures

## Abstract

The ancient mitochondrial genome of an Asiatic ibex (*Capra sibirica*) from Tangchaodun Ruins was obtained by high throughput sequencing. The observed damage pattern confirms the authenticity and reliability of the sequence. This mitogenome has a length of 16,583 bp, encompassing 13 protein-coding genes, 22 transfer RNAs, two ribosomal RNAs, one L-strand replication origin, and one control region. The total base composition of the mitochondrial genome is 31.94% A, 25.62% T, 12.48% G, 25.08% C, and 4.88% missing data with an AT composition of 57.56%. A maximum-likelihood phylogenetic tree based on the mitogenomes was recovered including other sequences of the genus *Capra* under the HKY+I + G4 model. Molecular evidence confirms the northern *Capra sibirica* lineage existed here ≥650 years ago and persists, with close genetic affinity to modern conspecifics.

## Introduction

The Asiatic ibex (*Capra sibirica* Pallas 1776) (Artiodactyla: Bovidae: Caprinae: *Capra*) (Ahmad et al. 2022), is a wild relative of the domestic goat (*Capra hircus*), and is placed in the genus *Capra* together with the West Caucasian tur (*Capra caucasica*), East Caucasian tur (*Capra cylindricornis*), Alpine ibex (*Capra ibex*), Markhor (*Capra falconeri*), Nubian ibex (*Capra nubiana*), Walia ibex (*Capra walie*), and Spanish ibex (*Capra pyrenaica*) (Nomura et al. 2013; Daly et al. 2022). One of the most distinctive features of the Asiatic ibex is that horns are found in individuals of both sexes (Fedosenko and Blank 2001). Males stand out with exceptionally well-developed horns, which are long, shaped like curved scimitars, and extend in a backward direction (Fedosenko and Blank 2001). The Asiatic ibex ranges across Central Asia to South Asia, including Afghanistan, India, Kazakhstan, Kyrgyzstan, Mongolia, Pakistan, Russia, Tajikistan, and Uzbekistan (Khan et al. 2014). In China, it is mainly distributed in Inner Mongolia, Gansu, and Xinjiang (Khan et al. 2014). As an alpine inhabitant, it lives on bare rock areas or gravelly steep slopes at an altitude of 3000–6000 m (Zhuo et al. 2022). In recent years, a continuous decline in its population in China has been caused by the gradual deterioration of its living environment and extensive hunting by man (Khan et al. 2016). It is currently listed as National Second—Class Protected Animal in China’s *National List of Key Protected Wildlife* and categorized as Near Threatened (NT) on the *IUCN Red List* (Wang et al. 2023).

**Figure 1.**
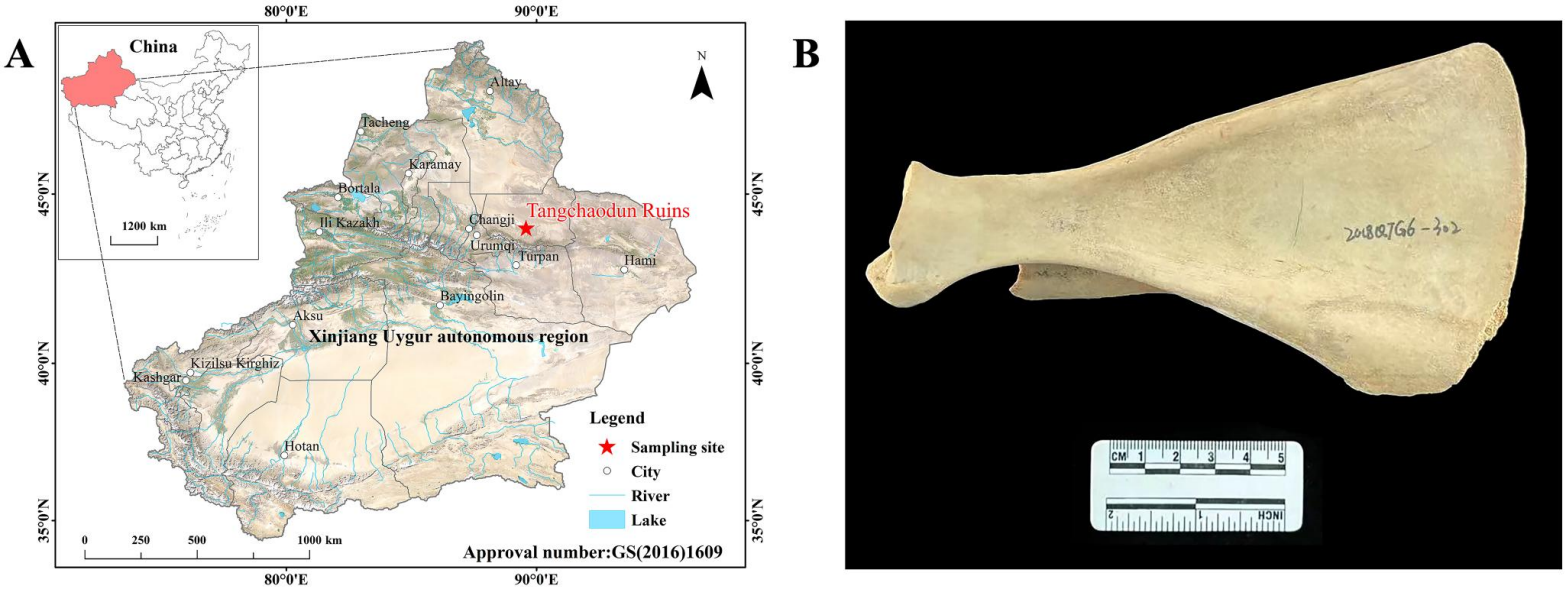
(A) Location of the Tangchaodun Ruins; (B) Ancient scapula remain analyzed in this study. Photographed by Guangjie Song.

The Tangchaodun Ruins (44°01’N, 89°35′E) are located in Qitai County, Xinjiang, China (Figure 1), serving as an important historical and geographical landmark in the northern part of the East Tianshan Mountains (Wei and Zheng 2022). The site was first constructed during the Zhenguan Period of the Tang Dynasty (627–649 CE), and continued to be used through the periods of the Gaochang Uyghur Kingdom (843–1275 CE), the Western Liao Dynasty (1124–1218 CE), and the Yuan Dynasty (1271–1368 CE), spanning multiple critical historical eras (Wei et al. 2023). As a key node of the Tianshan Corridor, the Tangchaodun Ruins historically had multiple functions: it was not only a hub for commercial exchanges on the Northern Route of the Silk Road, but also a military fortress for the central plains dynasties to govern the Western Regions (Ren and Wei 2021; 2022).

In this study, a scapula bone—a crucial diagnostic body part in zooarchaeology (Schmid 1972), whose morphological characteristics allow for the definitive identification of *Capra sibirica*—was obtained from the Tangchaodun Ruins. Using this bone, the mitochondrial genome of an ancient *Capra sibirica* individual was sequenced, assembled, and annotated (Figure 1). Our study provides valuable molecular data of an ancient *Capra sibirica* from this region, which could serve as an important reference for exploring its genetic continuity, but also provides reliable ancient mitochondrial genomic resources for further taxonomic, systematic, and evolutionary studies of the genus *Capra*.

## Materials and methods

The scapula from ancient *Capra sibirica* is housed at Jilin University (www.jlu.edu.cn, contact person: Dawei Cai, Email: caidw@jlu.edu.cn), assigned archaeological ID 2018QTG6-302 and lab code TCD06G. Based on the relics unearthed simultaneously with the skeletal remain, the dating of the skeletal remain can be determined to the Yuan Dynasty (Ren and Yu 2020). For sample preparation, an electric grinding tool and sterile disposable drills were used to remove the 2-mm outer bone surface. Following cleaning, specimens underwent treatment with 10% chloramine solution, decontamination with DEPC water, and a 5-minute immersion in 70% ethanol, then were dried under UV light. The next day, the specimen was ground into powder using an electric grinding tool, and 100 mg of each sample was collected. The ancient DNA extraction method in this study follows the protocol described by Dabney et al. (2013), utilizing the MinElute^®^ PCR Purification Kit for solution extraction. For library construction, the experimental approach developed by the Max Planck Institute in Germany was modified and optimized (Yates et al. 2021). After DNA library purification, paired-end sequencing (150 bp) was performed on an Illumina Hiseq X platform at Novogene Inc., Beijing, China.

The raw data was processed through the Paleomix pipeline (Schubert et al. 2014), employing the mitochondrial genome of *Capra sibirica* (GenBank accession: FJ207529) as the reference sequence. Mitochondrial coverage of the specimen’s mitogenome was assessed using Qualimap v2.2.1 (Okonechnikov et al. 2016), which had an average coverage of 11.85× (Supplemental Figure S1). The sequencing data of ancient DNA samples typically show a higher frequency of GC nucleotides at the 3′ and 5′ ends (Briggs et al. 2007). The terminal damage assessment was conducted using MapDamage v2.2.1 (Jónsson et al. 2013), which revealed the typical damage pattern associated with ancient DNA (Supplemental Figure S2). The consensus sequence in FASTA format was generated through ANGSD (Korneliussen et al. 2014). For the annotated mitochondrial genome, visualization was performed using the Proksee online server (Grant et al. 2023).

To clarify the phylogenetic relationship between the ancient sample and other members of the genus *Capra*, a dataset was analyzed comprising the TCD06G mitochondrial genome sequence, along with mitochondrial genome sequences of *Capra sibirica* (six), *Capra pyrenaica* (one), *Capra ibex* (one), *Capra walie* (one), *Capra nubiana* (one), *Capra cylindricornis* (two), *Capra caucasica* (one), *Capra aegagrus* (two), *Capra hircus* (two), and *Capra falconeri* (one), using *Pseudois nayaur* as an outgroup. A maximum-likelihood (ML) phylogenetic tree was obtained using the RAxML-ng program (Kozlov et al. 2019) under an unpartitioned HKY+I + G4 model, which was determined using ModelTest-NG v0.1.6 (Darriba et al. 2020). The mitochondrial DNA cytochrome b gene sequences (1095 bp) and control region sequences (760 bp) were respectively extracted from each sequence of the above mitochondrial genome dataset, with 60 additional mitochondrial DNA cytochrome b gene sequences and 43 additional control region sequences of modern *Capra sibirica* added for each. For the cytochrome b gene sequences, an ML phylogenetic tree was constructed using the RAxML-ng program under the HKY+G4 model, while for the control region sequences, an ML phylogenetic tree was built *via* the same program under the TPM1uf + I+G4 model. Phylogenetic trees were visualized using iTOL (Letunic and Bork 2021).

## Results

**Figure 2.**
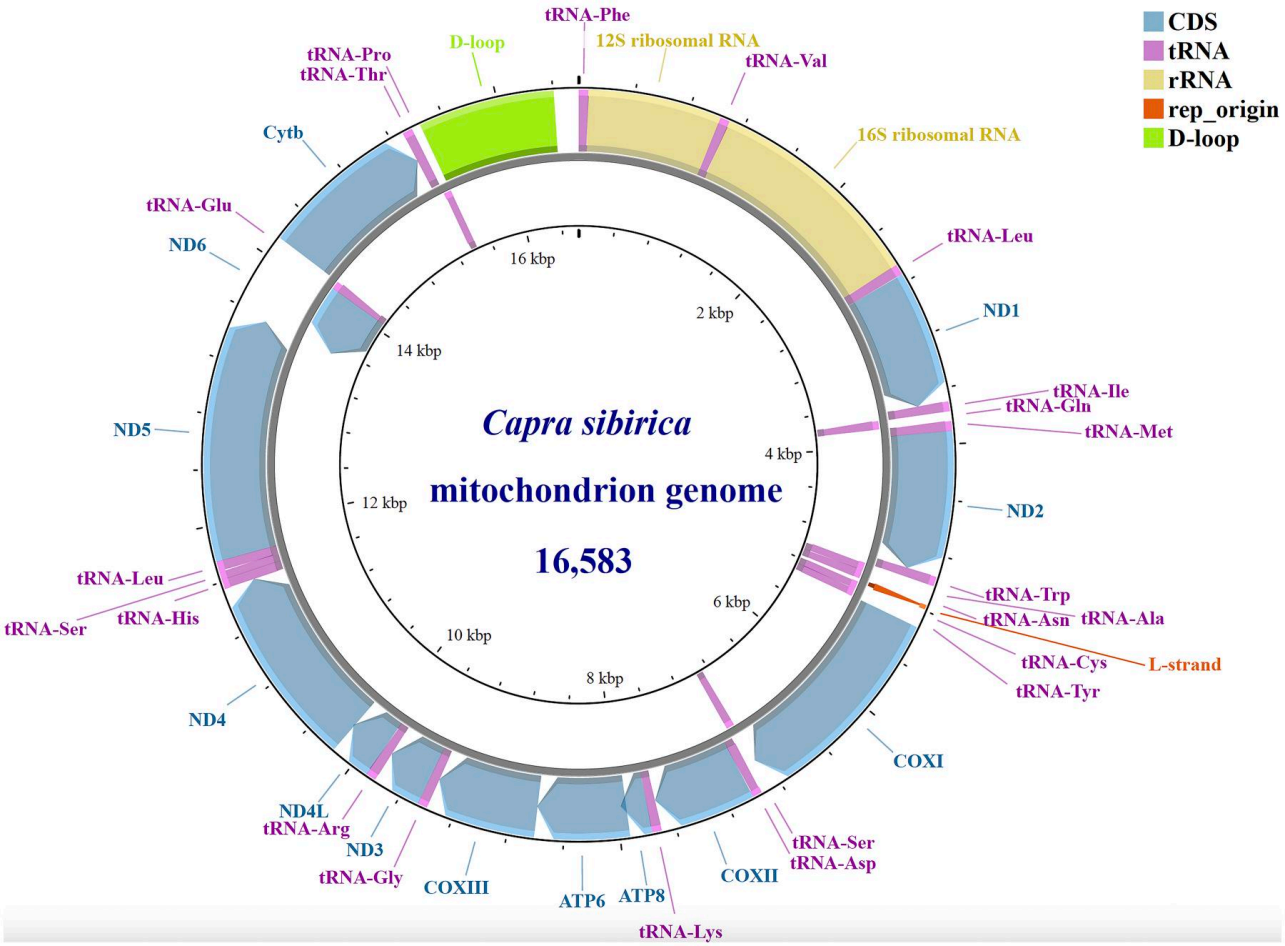
Mitochondrial gene map of an ancient *Capra sibirica* specimen from Tangchaodun Ruins (sample TCD06G; 16,583 bp).

The ancient mitochondrial genome sequence of specimen TCD06G (GenBank accession PV890828) had a total length of 16,583 bp, and it encompassed 13 protein-coding genes (PCGs), 22 transfer RNAs (tRNAs), two ribosomal RNAs (rRNAs) (12S rRNA and 16S rRNA), one replication origin (rep_origin) of the L-strand (light strand), and one control region (D-loop region). The base composition of the mitochondrial genome was 31.94% A, 25.62% T, 12.48% G, 25.08% C, and 4.88% N with an AT proportion of 57.56%. All protein-coding genes, except for the *ND6* gene, were transcribed in the same direction as the H-strand (heavy strand). Additionally, 14 out of the 22 tRNAs and the two rRNAs were also encoded on the H-strand (Figure 2). The lengths of the 12S rRNA, 16S rRNA, D-loop region, and rep_origin were 957 bp, 1,573 bp, 975 bp, and 32 bp, respectively. The 22 tRNAs ranged from 60 to 75 bp in length. The PCG *ND2*, *ND3*, and *ND5* started with ATA. ATG was used as the start codon for *ND4*, *ND4L*, *ND1*, *ND6*, *COX1*-*COX3*, *ATP6*, *ATP8*, and *CYTB*. Incomplete stop codons were found in *ND1*, *ND2*, *ND3*, *ND4*, and *COX3*, while complete stop codons were present in *ATP8*, *CYTB*, *ND5*, *ND6*, *COX1*, *COX2*, *ND4L*, and *ATP6*. In addition, due to the inherent DNA degradation of ancient samples, the assembled consensus sequences contain “N” (representing undetermined bases) corresponding to the gaps observed in the coverage plot (Supplemental Figure S1), which is a common technical characteristic of ancient DNA data.

**Figure 3.**
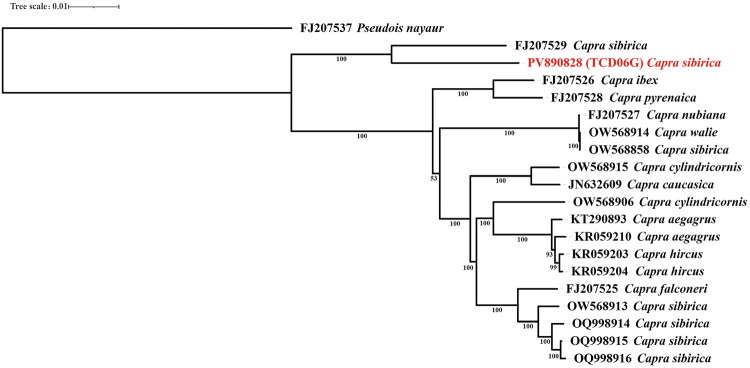
Maximum-likehood (ML) phylogenetic tree based on the mitogenome sequences of the genus *Capra*, with *Pseudois nayaur* functioning as an outgroup. Numbers at the branches represent bootstrap values with 1000 replications. The following sequences were used: FJ207537 *Pseudois nayaur* (unpublished), *Capra sibirica* PV890828 (TCD06G) (this study), FJ207529 *Capra sibirica* (Hassanin et al. 2009), FJ207528 *Capra pyrenaica* (Hassanin et al. 2009), FJ207526 *Capra ibex* (Hassanin et al. 2009), OW568914 *Capra walie* (Daly et al. 2022), OW568858 *Capra sibirica* (Daly et al. 2022), FJ207527 *Capra nubiana* (Hassanin et al. 2009), OW568915 *Capra cylindricornis* (Daly et al. 2022), JN632609 *Capra caucasica* (Hassanin et al. 2012), OW568906 *Capra cylindricornis* (Daly et al. 2022), KT290893 *Capra aegagrus* (unpublished), KR059210 *Capra aegagrus* (Colli et al. 2015), KR059204 *Capra hircus* (Colli et al. 2015), KR059203 *Capra hircus* (Colli et al. 2015), FJ207525 *Capra falconeri* (Hassanin et al. 2009), OW568913 *Capra sibirica* (Daly et al. 2022), OQ998914 *Capra sibirica* (unpublished), OQ998915 *Capra sibirica* (unpublished), OQ998916 *Capra sibirica* (unpublished).

The results of the maximum likelihood (ML) tree constructed based on the mitogenome sequences from all species within the genus *Capra* (Figure 3) showed that, unlike other species in the genus, *Capra sibirica* formed distinct clades. Specifically, TCD06G from this study forms a clade with one *Capra sibirica* sequence (FJ207529), which was separate from other sequences. Four *Capra sibirica* sequences (OW568913, OQ998914, OQ998915, OQ998916) clustered together and formed a sister clade with the branch composed of one *Capra falconeri* (FJ207525). In addition, one *Capra sibirica* sequence (OW568858) clustered together with two sequences of *Capra walie* (OW568914) and *Capra nubiana* (FJ207527), as has been shown to be consistent with nuclear genome study (Daly et al. 2022).

The results of the ML phylogenetic trees constructed separately based on the mitochondrial DNA cytochrome b gene sequences (Supplemental Figure S3) and control region sequences (Supplemental Figure S4) of all species within the genus *Capra* were consistent with those of the tree built using the mitogenomes, both showing that *Capra sibirica* formed distinct clades. Specifically, a subset of *Capra sibirica* individuals formed a sister clade with the branch containing an individual of *Capra falconeri*. In contrast, another subset of *Capra sibirica* individuals (including TCD06G from this study) exhibited a different clustering pattern, which echoes the clustering characteristic of TCD06G in the mitogenome-based tree.

## Discussion and conclusion

Molecular studies have not only identified two distinct maternal lineages within modern *Capra sibirica*—the northern and southern lineages—but have also debated their phylogenetic affinity, particularly whether they form sister clades or exhibit a non-sister relationship. Wang et al. (2023) noted that the southern lineage (primarily distributed in India, Tajikistan, and southern Xinjiang, China) and the northern lineage (ranging across Kazakhstan, Kyrgyzstan, Mongolia, Russia, and northern Xinjiang, China) show deep genetic divergence, with their phylogenetic placement suggesting they may not form a strict sister group; instead, this divergence was attributed to long-term geographical isolation driven by topographic barriers (e.g. the Pamir and Tianshan Mountains) and Pleistocene climate fluctuations, which restricted gene flow between the two lineages.

To contextualize the phylogenetic analyses of the ancient *Capra sibirica* individual (TCD06G) presented below, we first validated the reliability of the mitogenomic sequence data and characterized its biological variation—critical for ensuring subsequent phylogenetic inferences are robust to artifacts. Previous molecular biological research on the Asiatic ibex has focused on modern samples, while studies focusing on ancient ibex specimens remain relatively scarce; thus, verifying sequence integrity is particularly important for ancient material. For TCD06G, we conducted complementary analyses of its mitogenome: Firstly, we calculated the uncorrected p-distance between TCD06G and the modern *Capra sibirica* sequence (FJ207529), which was determined to be 0.03. Moreover, we counted the divergent sites between these two sequences, and the majority of these sites were localized to the 3rd codon positions. Notably, the variant sites exhibited a clear preference pattern of “3rd codon position > 1st codon position > 2nd codon position”—a distribution consistent with natural evolutionary processes. Collectively, these results confirm that the observed genetic differences between the sequences are characteristics of natural mutations rather than signs of random postmortem damage. This finding implies TCD06G and modern *Capra sibirica* share similar genetic backgrounds and exhibit stable genetic architectures, laying a reliable foundation for subsequent phylogenetic analysis of historical and contemporary lineages.

In our study, the phylogenetic trees further clarified the placement of the ancient *Capra sibirica* individual: TCD06G clusters specifically within the northern lineage of *Capra sibirica*, rather than grouping with the southern lineage or forming an independent branch between the two. This placement aligns with the geographical location of the Tangchaodun Ruins—an area within the modern distribution range of the northern lineage—and is consistent with Wang et al. (2023) observation that lineage-specific distribution patterns of *Capra sibirica* are strongly shaped by geography. This finding indicates that the northern lineage of *Capra sibirica* had already inhabited this region at least 650 years ago and has persisted there to the present day.

Furthermore, our phylogenetic analyses based on mitogenome sequences confirm that TCD06G is placed closer to a modern *Capra sibirica* sample with high node support—consistent with the sequence variation patterns observed earlier, and further validating their close genetic relationship. This consistency between sequence-level variation and phylogenetic placement reinforces the continuity of genetic traits across historical and contemporary *Capra sibirica* lineages.

## Supplementary Material

Supplementary Figures R4.pdf

## Data Availability

The genome sequence data that support the findings of this study are openly available in GenBank of NCBI at (https://www.ncbi.nlm.nih.gov/) under accession NO. PV890828. The associated BioProject, SRA, and Bio-Sample numbers are PRJNA1286915, SRR34377196, and SAMN49801778, respectively.
