# Supplementary material for: The ancient mitochondrial genome of Asiatic ibex (Capra sibirica) from Tangchaodun Ruins in Xinjiang, China, and its phylogenetic relationship
Source: Mitochondrial DNA B Resour. 2025 Dec 18;11(1):116–20. doi: 10.1080/23802359.2025.2604864 (PMC12720616; doi:10.1080/23802359.2025.2604864)
Supplement: Supplementary Figures R4.pdf [file TMDN_A_2604864_SM0212.pdf]

The ancient mitochondrial genome of Siberian ibex (*Capra sibirica*) from Tangchaodun Ruins in Xinjiang, China, and its phylogenetic relationship

Guangjie Song[1] Xinyan Zhang[2] Xiaohong Yu[1] Dawei Cai[2*]

1. School of Ethnic Studies, Xizang Minzu University, Xianyang, China
2. Bioarchaeology Laboratory, Jilin University, Changchun, China

✉Dawei Cai

caidw@jlu.edu.cn ORCID 0000-0001-6650-0217

[2] Bioarchaeology Laboratory, Jilin University, Changchun, China

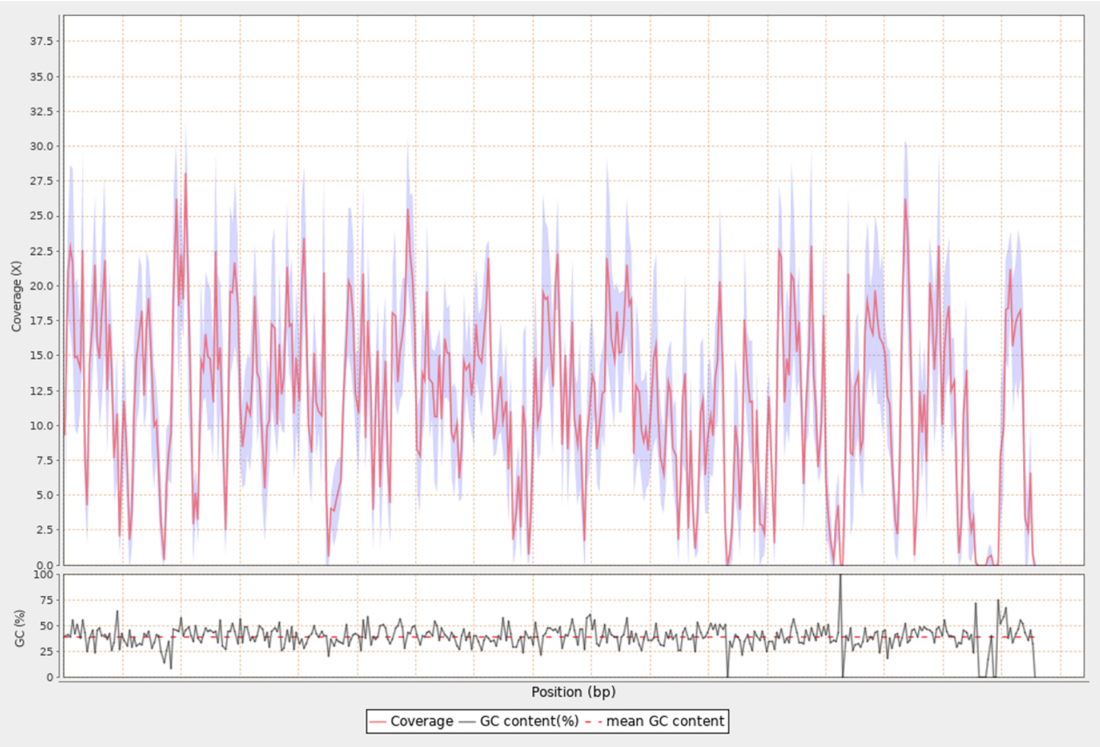

Figure S1. Coverage across reference about TCD06G.

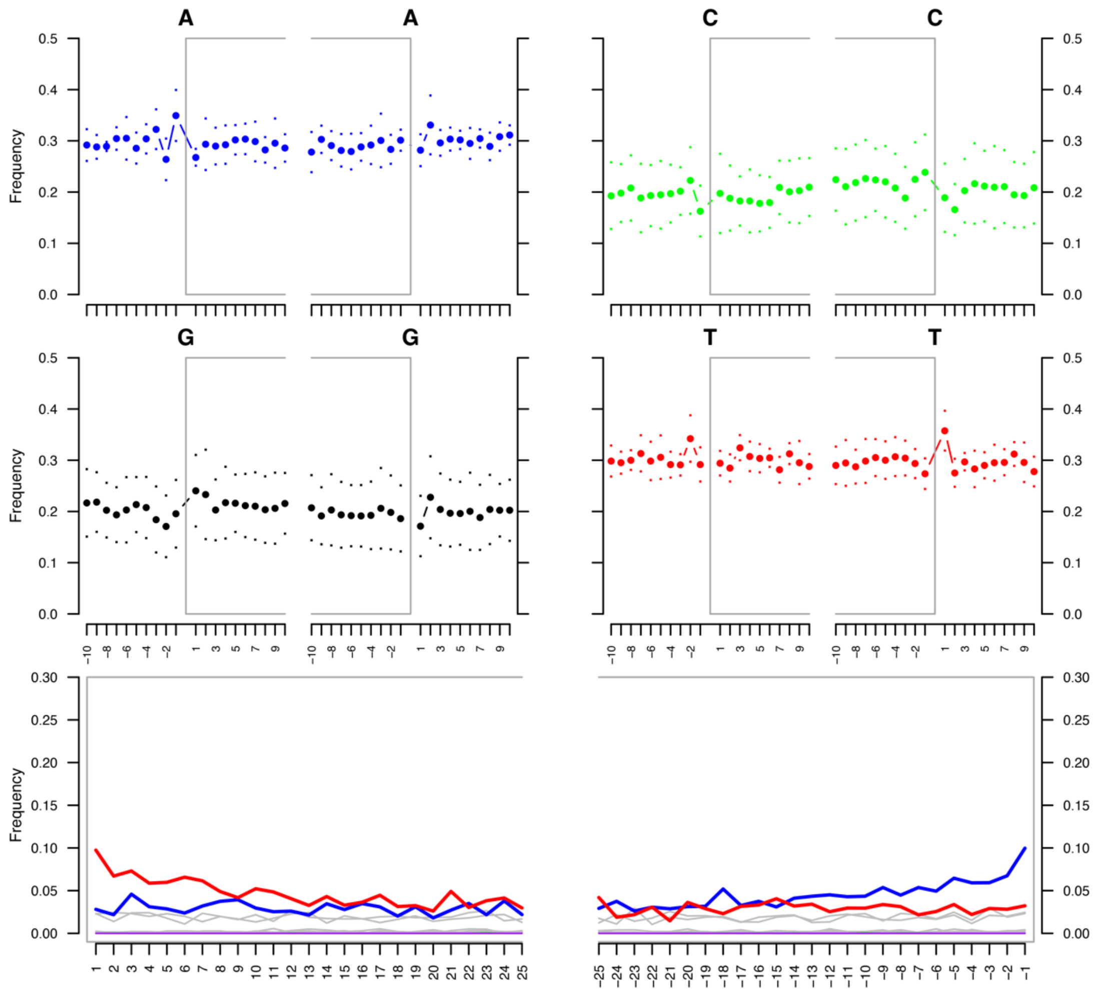

Figure S2. Mitochondrial DNA damage plots for TCD06G.

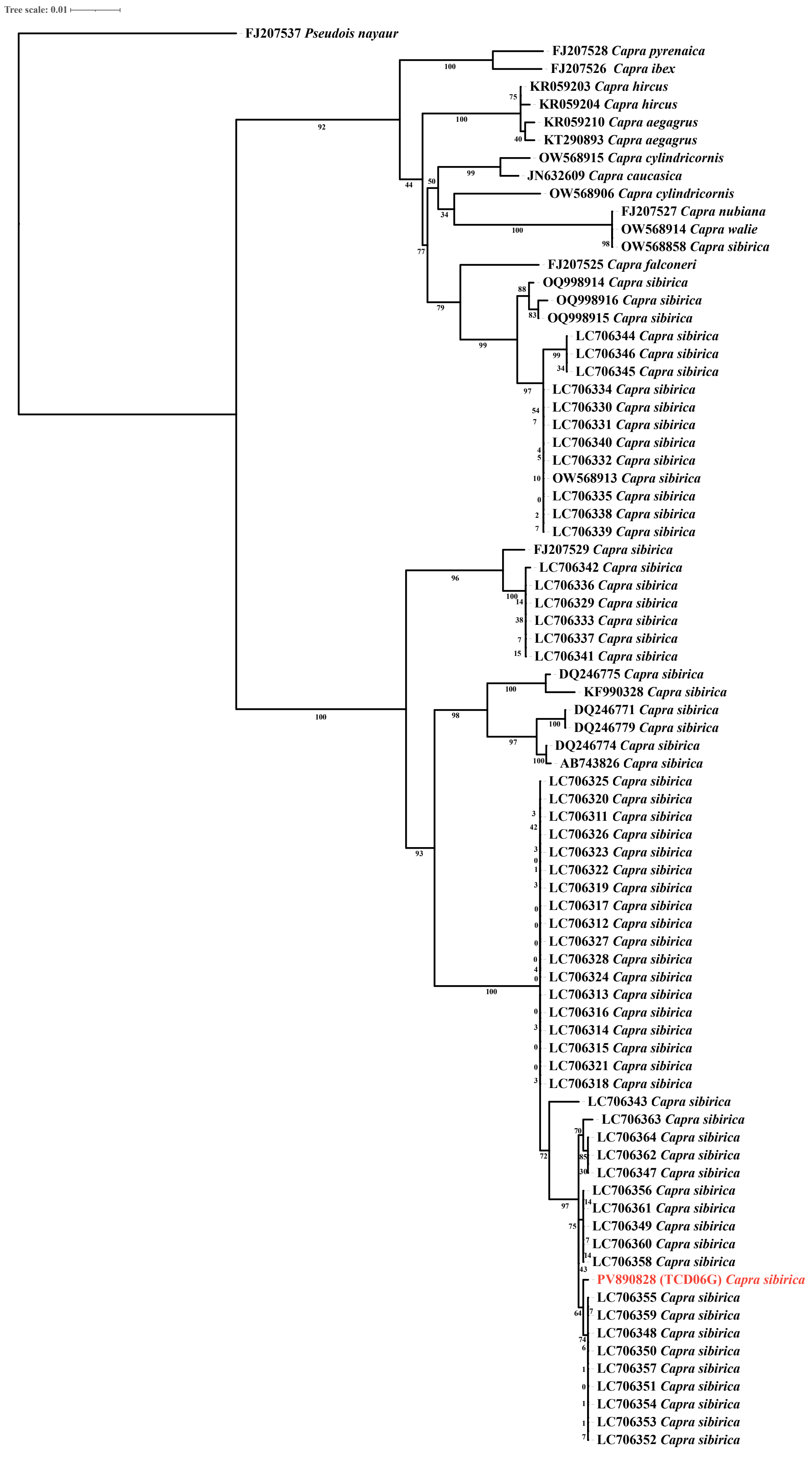

Tree scale: 0.01
FJ207537 Pseudois nayaur
FJ207528 Capra pyrenaica
FJ207526 Capra ibex
KR059203 Capra hircus
KR059204 Capra hircus
KR059210 Capra aegagrus
KT290893 Capra aegagrus
OW568915 Capra cylindricornis
JN632609 Capra caucasica
OW568906 Capra cylindricornis
FJ207527 Capra nubiana
OW568914 Capra walie
OW568858 Capra sibirica
FJ207525 Capra falconeri
OQ998914 Capra sibirica
OQ998916 Capra sibirica
OQ998915 Capra sibirica
LC706344 Capra sibirica
LC706346 Capra sibirica
LC706345 Capra sibirica
LC706334 Capra sibirica
LC706330 Capra sibirica
LC706331 Capra sibirica
LC706340 Capra sibirica
LC706332 Capra sibirica
OW568913 Capra sibirica
LC706335 Capra sibirica
LC706338 Capra sibirica
LC706339 Capra sibirica
FJ207529 Capra sibirica
LC706342 Capra sibirica
LC706336 Capra sibirica
LC706329 Capra sibirica
LC706333 Capra sibirica
LC706337 Capra sibirica
LC706341 Capra sibirica
DQ246775 Capra sibirica
KF990328 Capra sibirica
DQ246771 Capra sibirica
DQ246779 Capra sibirica
DQ246774 Capra sibirica
AB743826 Capra sibirica
LC706325 Capra sibirica
LC706320 Capra sibirica
LC706311 Capra sibirica
LC706326 Capra sibirica
LC706323 Capra sibirica
LC706322 Capra sibirica
LC706319 Capra sibirica
LC706317 Capra sibirica
LC706312 Capra sibirica
LC706327 Capra sibirica
LC706328 Capra sibirica
LC706324 Capra sibirica
LC706313 Capra sibirica
LC706316 Capra sibirica
LC706314 Capra sibirica
LC706315 Capra sibirica
LC706321 Capra sibirica
LC706318 Capra sibirica
LC706343 Capra sibirica
LC706363 Capra sibirica
LC706364 Capra sibirica
LC706362 Capra sibirica
LC706347 Capra sibirica
LC706356 Capra sibirica
LC706361 Capra sibirica
LC706349 Capra sibirica
LC706360 Capra sibirica
LC706358 Capra sibirica
PV890828 (TCD06G) Capra sibirica
LC706355 Capra sibirica
LC706359 Capra sibirica
LC706348 Capra sibirica
LC706350 Capra sibirica
LC706357 Capra sibirica
LC706351 Capra sibirica
LC706354 Capra sibirica
LC706353 Capra sibirica
LC706352 Capra sibirica

Figure S3. Maximum-likehood (ML) phylogenetic tree based on the mitochondrial DNA cytochrome b gene sequences (1095 bp) of genus *Capra*, with *Pseudois nayaur* functioning as an outgroup. Numbers at the branches representing the bootstrap values with 1,000 replications. The following sequences were used: FJ207537 *Pseudois nayaur* (unpublished), *Capra sibirica* PV890828 (TCD06G) (this study), FJ207529 *Capra sibirica* (Hassanin et al. 2009), FJ207528 *Capra pyrenaica* (Hassanin et al. 2009), FJ207526 *Capra ibex* (Hassanin et al. 2009), OW568914 *Capra walie* (Daly et al. 2022), OW568858 *Capra sibirica* (Daly et al. 2022), FJ207527 *Capra nubiana* (Hassanin et al. 2009), OW568915 *Capra cylindricornis* (Daly et al. 2022), JN632609 *Capra caucasica* (Hassanin et al. 2012), OW568906 *Capra cylindricornis* (Daly et al. 2022), KT290893 *Capra aegagrus* (unpublished), KR059210 *Capra aegagrus* (Colli et al. 2015), KR059204 *Capra hircus* (Colli et al. 2015), KR059203 *Capra hircus* (Colli et al. 2015), FJ207525 *Capra falconeri* (Hassanin et al. 2009), OW568913 *Capra sibirica* (Daly et al. 2022), OQ998914 *Capra sibirica* (unpublished), OQ998915 *Capra sibirica* (unpublished), OQ998916 *Capra sibirica* (unpublished), LC706344 *Capra sibirica* (Wang et al. 2023), LC706346 *Capra sibirica* (Wang et al. 2023), LC706345 *Capra sibirica* (Wang et al. 2023), LC706334 *Capra sibirica* (Wang et al. 2023), LC706330 *Capra sibirica* (Wang et al. 2023), LC706331 *Capra sibirica* (Wang et al. 2023), LC706340 *Capra sibirica* (Wang et al. 2023), LC706332 *Capra sibirica* (Wang et al. 2023), LC706335 *Capra sibirica* (Wang et al. 2023), LC706338 *Capra sibirica* (Wang et al. 2023), LC706339 *Capra sibirica* (Wang et al. 2023), LC706342 *Capra sibirica* (Wang et al. 2023), LC706336 *Capra sibirica* (Wang et al. 2023), LC706329 *Capra sibirica* (Wang et al. 2023), LC706333 *Capra sibirica* (Wang et al. 2023), LC706337 *Capra sibirica* (Wang et al. 2023), LC706341 *Capra sibirica* (Wang et al. 2023), DQ246775 *Capra sibirica* (unpublished), KF990328 *Capra sibirica* (unpublished), DQ246771 *Capra sibirica* (unpublished), DQ246779 *Capra sibirica* (unpublished), DQ246774 *Capra sibirica* (unpublished), AB743826 *Capra sibirica* (Nomura et al. 2013), LC706325 *Capra sibirica* (Wang et al. 2023), LC706320 *Capra sibirica* (Wang et al. 2023), LC706311 *Capra sibirica* (Wang et al. 2023), LC706326 *Capra sibirica* (Wang et al. 2023), LC706323 *Capra sibirica* (Wang et al. 2023), LC706322 *Capra sibirica* (Wang et al. 2023), LC706319 *Capra sibirica* (Wang et al. 2023), LC706317 *Capra sibirica* (Wang et al. 2023), LC706312 *Capra sibirica* (Wang et al. 2023), LC706327 *Capra sibirica* (Wang et al. 2023), LC706328 *Capra sibirica* (Wang et al. 2023), LC706324 *Capra sibirica* (Wang et al. 2023), LC706313 *Capra sibirica* (Wang et al. 2023), LC706316 *Capra sibirica* (Wang et al. 2023), LC706314 *Capra sibirica* (Wang et al. 2023), LC706315 *Capra sibirica* (Wang et al. 2023), LC706321 *Capra sibirica* (Wang et al. 2023), LC706318 *Capra sibirica* (Wang et al. 2023), LC706343 *Capra sibirica* (Wang et al. 2023), LC706363 *Capra sibirica* (Wang et al. 2023), LC706364 *Capra sibirica* (Wang et al. 2023), LC706362 *Capra sibirica* (Wang et al. 2023), LC706347 *Capra sibirica* (Wang et al. 2023), LC706356 *Capra sibirica* (Wang et al. 2023), LC706361 *Capra sibirica* (Wang et al. 2023), LC706349 *Capra sibirica* (Wang et al. 2023), LC706360 *Capra sibirica* (Wang et al. 2023), LC706358 *Capra sibirica* (Wang et al. 2023), LC706355 *Capra sibirica* (Wang et al. 2023), LC706359 *Capra sibirica* (Wang et al. 2023), LC706348 *Capra sibirica* (Wang et al. 2023), LC706350 *Capra sibirica* (Wang et al. 2023), LC706357 *Capra sibirica* (Wang et al. 2023), LC706351 *Capra sibirica* (Wang et al. 2023), LC706354 *Capra sibirica* (Wang et al. 2023), LC706353 *Capra sibirica* (Wang et al. 2023), LC706352 *Capra sibirica* (Wang et al. 2023).

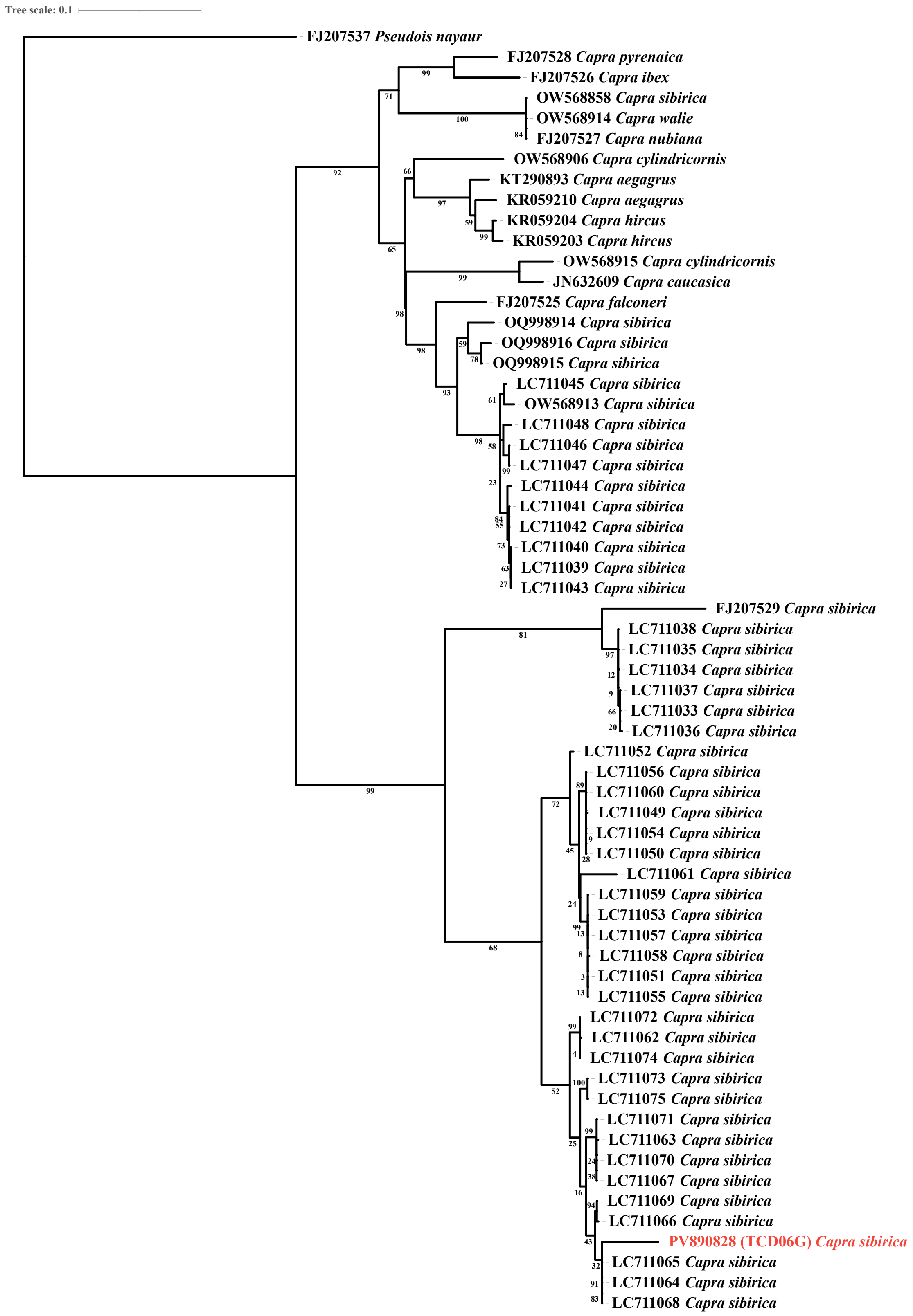

Figure S4. Maximum-likehood (ML) phylogenetic tree based on the mitochondrial DNA control region sequences (760 bp) of genus *Capra*, with *Pseudois nayaur* functioning as an outgroup. Numbers at the branches representing the bootstrap values with 1,000 replications. The following sequences were used: FJ207537 *Pseudois nayaur* (unpublished), *Capra sibirica* PV890828 (TCD06G) (this study), FJ207529

Capra sibirica (Hassanin et al. 2009), FJ207528 *Capra pyrenaica* (Hassanin et al. 2009), FJ207526 *Capra ibex* (Hassanin et al. 2009), OW568914 *Capra walie* (Daly et al. 2022), OW568858 *Capra sibirica* (Daly et al. 2022), FJ207527 *Capra nubiana* (Hassanin et al. 2009), OW568915 *Capra cylindricornis* (Daly et al. 2022), JN632609 *Capra caucasica* (Hassanin et al. 2012), OW568906 *Capra cylindricornis* (Daly et al. 2022), KT290893 *Capra aegagrus* (unpublished), KR059210 *Capra aegagrus* (Colli et al. 2015), KR059204 *Capra hircus* (Colli et al. 2015), KR059203 *Capra hircus* (Colli et al. 2015), FJ207525 *Capra falconeri* (Hassanin et al. 2009), OW568913 *Capra sibirica* (Daly et al. 2022), OQ998914 *Capra sibirica* (unpublished), OQ998915 *Capra sibirica* (unpublished), OQ998916 *Capra sibirica* (unpublished), LC711045 *Capra sibirica* (Wang et al. 2023), LC711048 *Capra sibirica* (Wang et al. 2023), LC711046 *Capra sibirica* (Wang et al. 2023), LC711047 *Capra sibirica* (Wang et al. 2023), LC711044 *Capra sibirica* (Wang et al. 2023), LC711041 *Capra sibirica* (Wang et al. 2023), LC711042 *Capra sibirica* (Wang et al. 2023), LC711040 *Capra sibirica* (Wang et al. 2023), LC711039 *Capra sibirica* (Wang et al. 2023), LC711043 *Capra sibirica* (Wang et al. 2023), LC711038 *Capra sibirica* (Wang et al. 2023), LC711035 *Capra sibirica* (Wang et al. 2023), LC711034 *Capra sibirica* (Wang et al. 2023), LC711037 *Capra sibirica* (Wang et al. 2023), LC711033 *Capra sibirica* (Wang et al. 2023), LC711036 *Capra sibirica* (Wang et al. 2023), LC711052 *Capra sibirica* (Wang et al. 2023), LC711056 *Capra sibirica* (Wang et al. 2023), LC711060 *Capra sibirica* (Wang et al. 2023), LC711049 *Capra sibirica* (Wang et al. 2023), LC711054 *Capra sibirica* (Wang et al. 2023), LC711050 *Capra sibirica* (Wang et al. 2023), LC711061 *Capra sibirica* (Wang et al. 2023), LC711059 *Capra sibirica* (Wang et al. 2023), LC711053 *Capra sibirica* (Wang et al. 2023), LC711057 *Capra sibirica* (Wang et al. 2023), LC711058 *Capra sibirica* (Wang et al. 2023), LC711051 *Capra sibirica* (Wang et al. 2023), LC711055 *Capra sibirica* (Wang et al. 2023), LC711072 *Capra sibirica* (Wang et al. 2023), LC711062 *Capra sibirica* (Wang et al. 2023), LC711074 *Capra sibirica* (Wang et al. 2023), LC711073 *Capra sibirica* (Wang et al. 2023), LC711075 *Capra sibirica* (Wang et al. 2023), LC711071 *Capra sibirica* (Wang et al. 2023), LC711063 *Capra sibirica* (Wang et al. 2023), LC711070 *Capra sibirica* (Wang et al. 2023), LC711067 *Capra sibirica* (Wang et al. 2023), LC711069 *Capra sibirica* (Wang et al. 2023), LC711066 *Capra sibirica* (Wang et al. 2023), LC711065 *Capra sibirica* (Wang et al. 2023), LC711064 *Capra sibirica* (Wang et al. 2023), LC711068 *Capra sibirica* (Wang et al. 2023).
